# The degree of doneness affected molecular changes and protein digestibility of pork

**DOI:** 10.3389/fnut.2022.1084779

**Published:** 2023-01-04

**Authors:** Yu Han, Hui Liu, Qian Li, Di Zhao, Kai Shan, Weixin Ke, Miao Zhang, Chunbao Li

**Affiliations:** ^1^Key Laboratory of Meat Processing and Quality Control, MOE, College of Food Science and Technology, Nanjing Agricultural University, Nanjing, China; ^2^Key Laboratory of Meat Processing, MARA, College of Food Science and Technology, Nanjing Agricultural University, Nanjing, China; ^3^Jiangsu Collaborative Innovation Center of Meat Production, Processing and Quality Control, College of Food Science and Technology, Nanjing Agricultural University, Nanjing, China

**Keywords:** doneness gradient, molecular dynamics simulation, myofibrillar proteins, structure, digestibility

## Abstract

The degree of doneness has been shown to have a great impact on eating quality of meat, however, it is little known whether it affects protein digestibility of meat. In this study, we explored molecular changes and protein digestibility of pork under different degree of doneness. Pork chops were cooked in a 100°C water bath for about 26 min and a gradient decrease in doneness was obtained from outer to inner layers of samples. Compared with the raw samples, the cooked samples’ active and total sulfhydryl contents, surface hydrophobicity, and turbidity increased but its solubility decreased. The inner layers with lower doneness contained higher α-helix, and fluorescence intensities of tryptophan and tyrosine residues than the outer layers with higher doneness. The pepsin and pancreatin digestibility of meat proteins in the inner layers were higher than those of the outer layers. Molecular simulation analysis showed that the most abundant protein in pork, i.e., myosin in the outer layers were more stable with an increased number of hydrogen bonds, making it difficult to be digested. These findings provided a new insight into the heterogeneity of meat nutritional quality due to the existence of doneness gradient.

## Graphical Abstract

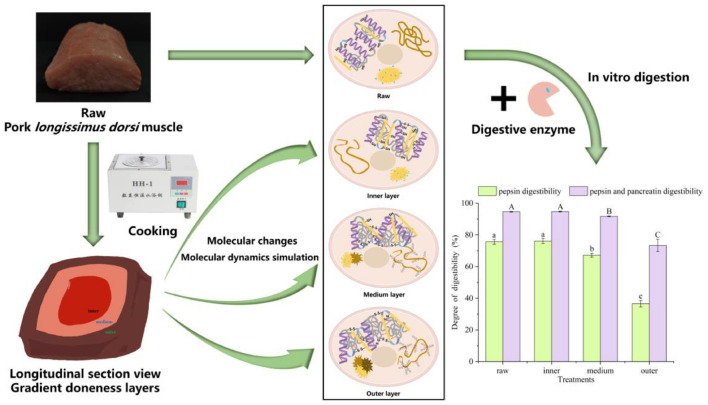

## Highlights

- The heterogeneity of nutritional quality of meat due to the existence of doneness gradient.
- The digestibility of meat protein in the inner layer was higher than middle and outer layers.
- The changes of hydrophobic interaction and hydrogen bonds affected protein structure.
- The changes of different doneness layer myofibrillar protein structures affected protein digestibility.

## 1 Introduction

Meat and meat products are vital source of high-quality protein, fatty acids, iron, zinc, and B vitamins, which have played a vital role in human evolution (1). Heating is an important process for preparing edible meat during which protein denaturation occurs and has a great effect on meat quality (2). There are many different heat methods, including frying, baking, and stewing, most of which mainly rely on heat conduction, and the heating rate is slow, causing a decreasing temperature or doneness gradient from the outer to the inner. This may cause differences in protein digestibility and other meat quality attributes (3).

Myofibrillar proteins (MPs) account for 50–60% of the whole meat protein (4). In addition, the MPs have vital functional characteristics closely related to water retention, gelation, rheology and sensory quality. Generally speaking, the process of meat applied to cooking may induce great changes in MPs affecting the functionality, digestibility and nutrition of meat. During processing, heating temperature affects the structure of MPs, causing contraction of the myosin, exposure of the hydrophobic sites, and aggregation of proteins. This will make the protein structure more compact, and further affect the digestibility of meat protein to a certain extent (5). Kaur et al. (6) found that the sarcomere structure of myofibril was obviously destroyed after pepsin digestion of raw meat, but it kept relatively intact in cooked meat. Myosin is the main component of myofibrillar filament, and its structural changes will play a critical role.

The excessive oxidation caused by heat processing will induce aggregation and cross-linking, modify the active site, and reduce the digestibility of protein and the bioavailability of amino acids, which have a negative influence on the nutritional values of meat (7). In the process of heat treatment, the temperature shows a gradient decline from the outer to the inner layers, which may induce changes in the secondary structure and related enzyme active sites of meat proteins, and affect the digestion efficiency and digestion rate of protein (8). The protein digestion efficiency and rate will further affect the absorption and utilization of dietary protein within human body (9). However, few researches have paid attention to the structural changes of MPs induced by temperature gradient and its effect on digestibility.

In this research, we explored the effects of temperature gradient (the degree of doneness) on pork MPs structure, surface hydrophobicity and meat digestibility. Molecular dynamics simulation (MDS) was taken to further explore the underlying mechanism in conformational changes of pork myosin under the different degree of doneness.

## 2 Materials and methods

### 2.1 Chemicals

BCA protein assay kit was purchased from Thermo Scientific (Rockford, IL, USA). Pepsin and pancreatin were purchased from Sigma Aldrich (St. Louis, MO, USA). Broad pre-stained protein marker, sample buffer, LDS sample buffer and 4–20% precast gels were purchased from GenScript (Piscataway, NJ, USA).

### 2.2 Sample preparations

**FIGURE 1:**
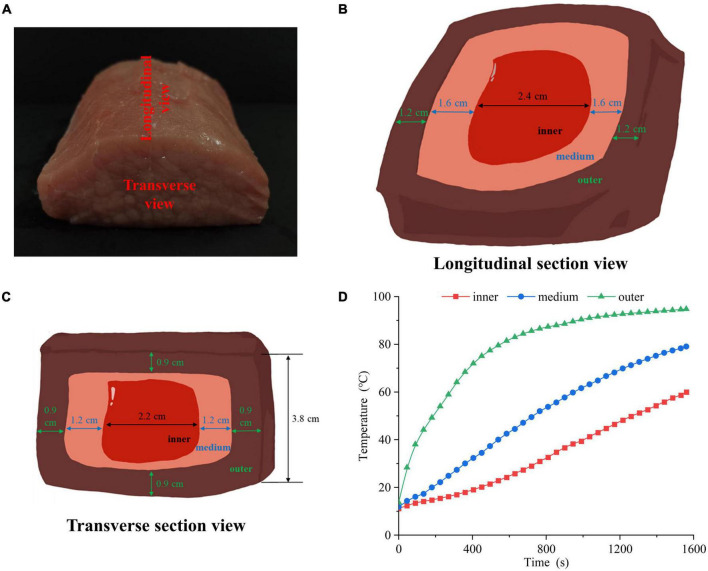
**(A)** Physical picture of cutting the pork *longissimus dorsi* muscle. The **(B)** longitudinal section view, **(C)** transverse section view and **(D)** heating time curves of pork samples in different doneness layers.

The pork *longissimus dorsi* muscle was purchased from Sushi Meat Co., Ltd (Jiangsu, China). As shown in Figure 1A, each muscle was cut into 10 cm × 8 cm × 5 cm chops and the length scale of 10 cm was along the muscle fiber direction. All connective tissue and visible fat were removed. All pork chops were put in polyethylene bags separately and a portable thermometer was inserted. Then, the chops were cooked in a 100°C water bath until 60°C in the center of pork chops. After cooking, the chops were moved from the water bath, and cooled in ice bath for 15 min. The cooled samples were cut into three portions: outer, middle, and inner layers. Figures 1B, C showed the longitudinal and transverse section views of the sample, respectively. The longitudinal section was parallel to the muscle fiber direction, and the transverse section was perpendicular to the muscle fiber direction. Four groups were set up, including raw meat (4°C, raw), inner, middle and outer layers. The inner, middle and outer temperatures were recorded (Figure 1D).

### 2.3 MPs extraction

The MPs were extracted by the method of Xu et al. (10) with minor modifications. Briefly, the samples were put in a meat blender at a speed of 3,000 rpm for 30 s (GM 200, Retsch, Germany). The meat pastes were then homogenized in buffer (0.1 mol/L KCl, 20 mmol/L K_2_HPO_4_/KH_2_PO_4_, 1 mmol/L ethyleneglycoltetraacetic acid, and 2 mmol/L MgCl_2_, pH 7.0) at a ratio of 1:4 (w/v) using a homogenizer at 9,000 rpm for 1 min (PD500-TP, PRIMA, England). Then, the meat homogenate was separated through two layers gauze. The filtrate was centrifuged at 4°C, 2,000 *g* for 10 min, and the sediments were collected. The above steps were repeated three times. Then, the sediments were homogenized with 0.1 mol/L KCl solution (1:4, w/v) at 9,000 rpm for 30 s and centrifuged at 4°C, 2,500 *g* for 10 min. This step was repeated once. BCA protein assay kit (Thermo Scientific, Rockford, IL, USA) was taken to measure the protein content. The acquired protein was dispersed in phosphate-buffered saline (PBS, 0.6 mol/L KCl, 20 mmol/L K_2_HPO_4_/KH_2_PO_4_, pH 7.5) to a final concentration of 10 mg/ml.

### 2.4 *In vitro* digestion

The digestibility of samples was measured according to Brodkorb et al. (11) with minor modifications. Briefly, the meat samples (0.8 g) were dissolved in 3 ml simulated gastric fluid and the details are shown in Supplementary material.

### 2.5 Protein digestibility

The gastric and gastrointestinal digestion products were, respectively, added with anhydrous ethanol (1:3, v/v) and incubated at 4°C for 12 h (12). Then the mixture was centrifuged at 4°C, 10,000 *g* for 20 min to collect the sediments. The sediments were dissolved in protein extraction buffer. Then, the solution was centrifuged at 4°C, 4,000 *g* for 5 min to collect the supernatants. BCA protein assay kit was taken to measure protein content in the supernatants which was the protein content of the digestion products. The protein digestibility of meat was calculated as follows:


$$\text{Protein digestibility (\%)}=\frac{{\text{W}}_{0}-{\text{W}}_{1}}{{\text{W}}_{0}}\times 100$$

W_1_ and W_0_ are the protein content (g) in the sediment after gastric and gastrointestinal digestion and the meat sample before digestion, respectively.

### 2.6 Sodium dodecyl sulfate polyacrylamide gel electrophoresis (SDS-PAGE)

MPs (2 mg/ml) were dissolved in the sample buffer (GenScript, Piscataway, NJ, USA) or LDS sample buffer (GenScript, Piscataway, NJ, USA) at a ratio of 4:1 (v/v) and incubated at 95°C for 10 min. Then, mixture (15 μl) and broad pre-stained protein marker (8 μl, GenScript, Piscataway, NJ, USA) were loaded in each lane of the SDS-PAGE gels (GenScript, 4–20%, 15 wells). Electrophoresis was conducted by a Mini-Protean Tetra System (Bio-Rad Laboratories, Hercules, CA). The parameters of electrophoresis were set as 80 V for 30 min, followed by 100 V for 80 min at 4°C. After separation, gels were stained and images were acquired by an image scanner (GE Healthcare, Little Chalfont, SE).

### 2.7 Total sulfhydryl and active sulfhydryl group contents

Total sulfhydryl content was determined as follows. 5 ml 20 mmol/L Tris–HCl buffer (8 mol/L urea, 10 mmol/L ethylenediaminetetraacetic acid, pH 8.0) and 0.1 ml Ellman reagent (10 mmol/L, pH 8.0) were mixed with 0.5 ml MPs (1 mg/ml). The mixture was kept at 25°C in the dark for 1 h, and its absorbance at 412 nm was acquired. Active sulfhydryl content was determined with Tris–HCl buffer without 8 mol/L urea, other steps are the same as above. Sulfhydryl group contents were calculated as follows:


$$\text{Sulfhydryl content (nmol/mg protein)}=73.53\times {\text{A}}_{412}\times \frac{\text{D}}{\text{C}}$$

where A_412_ is the absorbance at 412 nm; C is protein content in mg protein/ml; and D is dilution factor equaling to 11.2.

### 2.8 Surface hydrophobicity

The fluorescence probe 8-Anilino-1-naphthalenesulfonic acid (ANS) was taken to measure the surface hydrophobicity of MPs (10). An ANS solution (15 mmol/L, pH 7.0) was dissolved in phosphate-buffered saline (PBS) and the details are shown in Supplementary material.

### 2.9 Turbidity

The turbidity was determined according to Jiang et al. (13) with some modifications. The MPs (2 mg/ml) were prepared by PBS. After equilibration for 20 min at room temperature, the absorbance at 340 nm was measured with PBS as a blank.

### 2.10 Protein solubility

To determine protein solubility, 5 ml MPs (2 mg/ml) were centrifuged at 4°C, 10,000 *g* for 20 min. Then, the supernatants and MPs protein contents were measured at 562 nm. The solubility of MPs was calculated as follows:


$$\begin{array}{l} \text{Protein solubility (\%)}\\ =\frac{\text{Protein content in supernatant solution}}{\text{Total protein content in protein sample}}\times 100 \end{array}$$

### 2.11 Raman spectroscopy

Raman spectroscopy of MPs (about 1 g) were acquired according to Jiang et al. (14) using a spectrometer (HR800, Horiba/Jobin Yvon, Longjumeau, France). Raman spectra were measured at 400–3,600 cm^–1^ with a grating of 300 g/mm and the integration time of 50 s. Each sample was measured three times and each spectrum was an average of three scans.

### 2.12 Fluorescence spectroscopy

Fluorescence spectroscopy of MPs were determined according to Khan et al. (15) with minor modifications and the details are shown in Supplementary material.

### 2.13 MDS

The protein changes of MPs from different degrees of doneness were analyzed by MDS. NAMD 2.12 and Charmm 27 Force Field softwares were taken to determine all MDS. The myosin structural file was obtained from the Protein Database (PDB ID: 2vas). Molecular structural changes of myosin at 4°C and temperature fitting curves were simulated to determine the impact of the degree of doneness on protein structure. The whole MDS process includes 1 ns of water balance, 1,000 steps of energy minimization, 1,000 ps of temperature relaxation, and 50 ns of classical MDS.

### 2.14 Statistical analysis

The experiment was repeated 6 times. For all statistical tests, the level of significance was set to 0.05 and the data were presented as means ± standard deviations. Differences among different degrees of doneness were analyzed by Duncan’s multiple-range comparisons in the context of analysis of variance model using the SAS 9.1.2 program (SAS Institute Inc., Cary, NC, USA). Figures were made using the Origin 2021.

## 3 Results and discussion

### 3.1 Digestibility of pork samples and functional properties of MPs with different degrees of doneness

**FIGURE 2:**
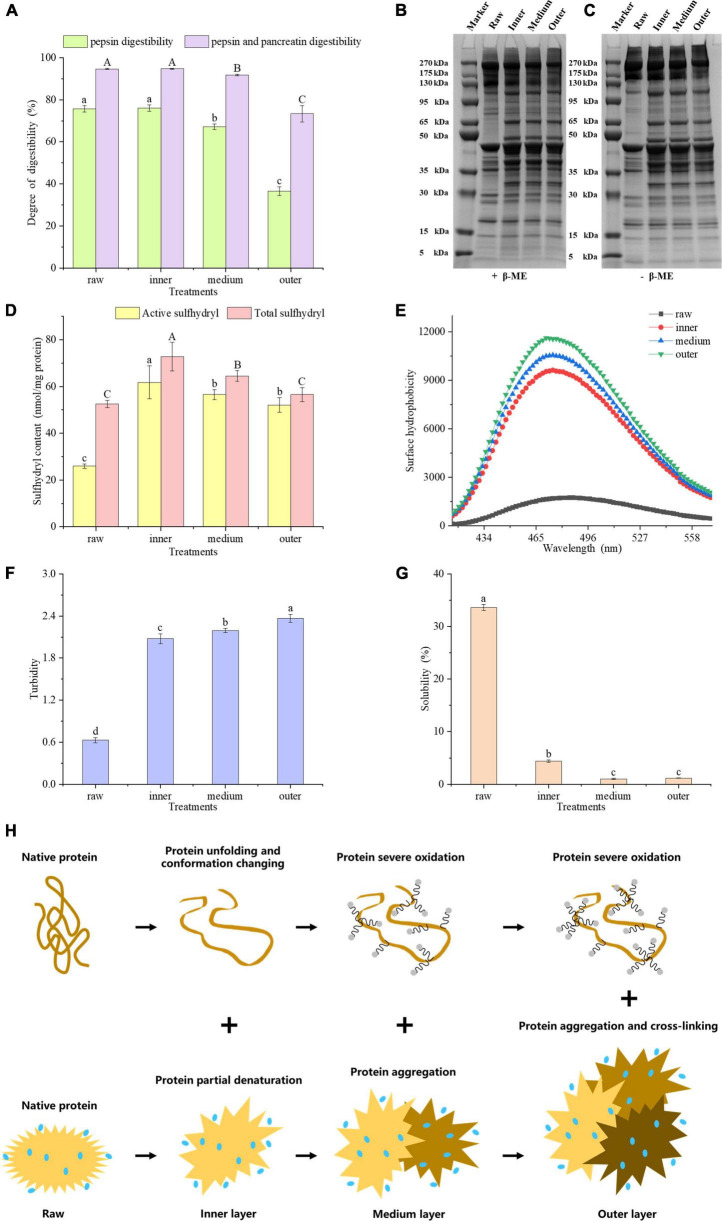
**(A)** Gastric and gastrointestinal digestibility of pork proteins from different layers. SDS-PAGE patterns of reduced **(B)** and unreduced **(C)** proteins. **(D)** Sulfhydryl content, **(E)** surface hydrophobicity, **(F)** turbidity, **(G)** solubility of MP samples in different layers. **(H)** Mechanistic diagram of MP conformation and aggregation changes of different layers. Letters A, B, C or a–d indicate significant differences in measured variables among different treatments (*P* < 0.05).

As shown in Figure 2A, the digestibility of meat proteins, including gastric and intestinal phase digestibility. The digestibility of pepsin and pancreatin in the inner layer was the highest, which were 76.12 and 94.76%, respectively. The pancreatin digestibility of meat protein in the middle and outer layers were significantly lower than that in raw samples by 2.88 and 21.27%, respectively (*P* < 0.05). The pepsin and pancreatin digestibility were significantly decreased from the inside to outside layers (*P* < 0.05), but no significant difference was found between the inner layer and raw samples (*P* > 0.05). The physical and chemical properties of meat protein considerably affect its digestibility (16). We hypothesized that the lower digestibility of samples could be linked to protein oxidation, denaturation, structural changes and aggregation (17).

On SDS-PAGE gels, 5% β-mercaptoethanol (β-ME) treated MPs showed lower band intensities for raw samples than for cooked samples (Figure 2B). The band intensities of MPs with high molecular weight, e.g., 175 – 270 kDa, 95 – 130 kDa, decreased from the inside to outside layers (Figure 2C). The band profiles of SDS-PAGE gels were greatly different before and after the β-ME treatment. The intensities of 35–50 kDa bands in β-ME treated groups were higher than those of untreated groups (Figures 2B, C). However, the intensities of higher molecular weight bands in β-ME treated groups were lower than those of untreated groups. These results indicated that proteins underwent oxidation with the generation of disulfide bonds during heat treatment. Comparisons of the band intensities of MPs among different layers indicated that the protein conformation of MPs could be altered from a closed conformation to an open conformation in the inner layer. And some sulfhydryl groups were exposed and oxidized to form disulfide bonds, while in outer layer, a large number of disulfide bonds were formed due to severe oxidation, and we speculated that the proteins may be cross-linked by disulfide bonds (18).

Total sulfhydryl group refers to whole sulfhydryl group content in a protein, counting those exposed on the protein surface network and those hidden in the protein. Zhang et al. (19) found that sulfhydryl groups in proteins, particularly active sulfhydryl groups, are facilely converted into disulfide bonds during processing and storage of meat. Generally speaking, when the sulfhydryl groups in proteins are exposed, they are facilely oxidized to disulfide bonds, bringing about a reduction in the content of sulfhydryl groups. Benjakul et al. (20) showed that the reduction of total sulfhydryl content may be relevant to the breakage of ordered protein structure. The improve of disulfide bonds always brings about a higher protein aggregation and lower digestibility of macromolecular proteins (21). Figure 2D showed that the contents of active and total sulfhydryl groups in the three layers were higher than those of raw samples, respectively. The exposure of sulfhydryl groups will contribute to the elevation of the content of active and total sulfhydryl groups. The total sulfhydryl content significantly decreased from the inside to outside layers (*P* < 0.05), and the active sulfhydryl content in middle and outer layers were greatly lower than that in inner layers (*P* < 0.05). We hypothesized that the decrease in active and total sulfhydryl contents of middle and outer layers may be attributed to the oxidation of exposed sulfhydryl groups. In this study, the sulfhydryl groups of MPs were transformed into disulfide bonds by heat-induced oxidation in the middle and outer layers, which is consistent with Zhang et al. (19).

Hydrophobic interaction is the most important stable force for keeping the tertiary structure of proteins. The fluorescence of ANS itself is weak, and when it binds to the hydrophobic domain in a protein, the fluorescence intensity improves obviously, accompanied by the blue shift of the maximum emission wavelength (22). ANS as a fluorescence probe can reflect the changes of protein tertiary structure (23). Heating may bring about the exposure of hydrophobic residues and increase the hydrophobicity of a protein. To characterize the hydrophobicity of MPs from different layers, we measured its fluorescence intensity at 475 nm. According to the Figure 2, the surface hydrophobicity of MPs improved from the inside to outside layers, indicating the exposure of hydrophobic residues and the expansion of protein structure. An increasing number of hydrophobic groups were exposed as the degree of doneness increased, which undermined the hydrophobic interaction in a protein and brought about an enhancement in protein surface hydrophobicity.

Turbidity plays a vital part in evaluating protein binding and aggregation. The turbidity values increased significantly from the inner to the outer layers (*P* < 0.05, Figure 2F). This may account for the increase in the degree of proteins denaturation and aggregation, which influences the scattering of light and interferes with the propagation of light, thereby increasing absorbance (24). Ekezie et al. (25) showed that the increase of turbidity may account for the unfolding and aggregation of protein molecules. According to the present study, we speculated that from the inner to the outer layers, MPs were partially denatured and unfolded at first, and its protein aggregation increased positively with the degree of doneness.

The solubility of protein refers to the amount or corresponding degree of protein dispersion in buffer, which is very susceptible to protein denaturation and aggregation. In contrast to the raw samples, the protein solubility of cooked samples reduced greatly (*P* < 0.05, Figure 2G). At the same time, the solubility of proteins from the middle layer and the outer layer was lower than that from the inner layer (*P* < 0.05), but there was no significant difference between the middle layer and the outer layer (*P* > 0.05). The extent of protein denaturation can be assessed by changes in protein dissolution. A reduction in solubility means the damage of protein structure (26). The current research showed that the solubility of MPs reduced with the degree of doneness, indicating that overheating treatment destroyed the protein structure and improved the degree of protein aggregation. From the inner to the outer layers, surface hydrophobicity and turbidity of MPs increased, but active sulfhydryl, total sulfhydryl content and protein solubility of MPs decreased. According to these results, we hypothesized that the protein molecular conformation could be partially expanded and some sulfhydryl groups would be oxidized to form disulfide bonds, and the buried hydrophobic groups could be exposed. As a result, the degree of protein aggregation improved, bringing about the decrease in protein digestibility.

Taken together, the proteins in inner doneness layers were partially denatured, causing the unfolding of the proteins and the changes in conformation. On the other hand, proteins in the middle and outer layers were oxidized seriously, causing protein aggregation and cross-linking (Figure 2H).

### 3.2 Secondary structure of MPs and MDS

**FIGURE 3:**
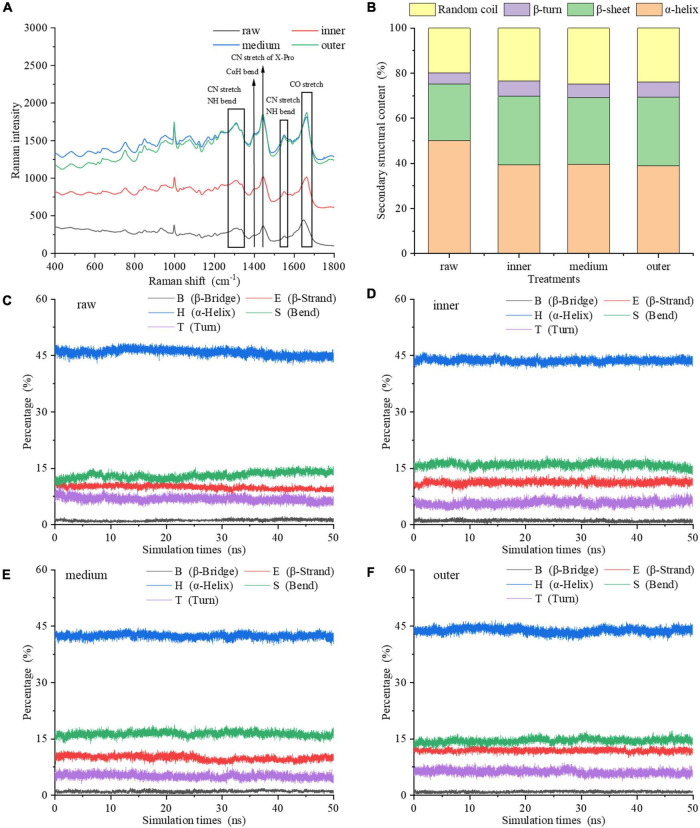
Secondary structure of MP samples from different doneness layers: **(A)** Raman spectra; **(B)** α-helix, β-sheet, β-turn, and random coil contents. **(C–F)** Changes of the main secondary structures of myosin molecules in raw sample, inner, medium and outer layers during the 50 ns MDS.

Due to the sensitivity of the vibration frequency to the hydrogen bond environment, the Raman spectra of the amide chain can be taken to represent the secondary structure (Figure 3A). For vibrations involving carbonyl and amide functional groups, there are strong Raman bands: amide I (predominantly C = O stretch), amide II (∼60% N-H bend +∼40% C-N stretch) and amide III (∼40% C-N stretch +∼30% N-H bend) modes (27). Except these amide modes, the amide IIp mode of proline and the amide S mode are also used in Raman spectroscopy (28, 29). The analysis of amide III (∼1,250–1,350 cm^–1^), amide S (∼1,390 cm^–1^), amide II (∼1,520–1,560 cm^–1^), and amide I (∼1,630–1,680 cm^–1^) regions reveals the relative contents of α-helix, β-sheet, β-turn and random coil secondary structure in proteins and peptides (28, 30). From the inner to the outer layers, the intensities of Raman spectra of MPs increased. Comparing the Raman spectra of MPs of raw samples with cooked samples in amide III band, it was found that the peak intensity changed, and the peak positions of three layers were red-shifted compared to the raw samples. As the most informative part of protein secondary structure, the spectrum of amide I region was taken to fit the curve, and the changes of secondary structure of MPs were further analyzed (31). The amide I band intensity of MPs was changed by the degree of doneness.

Figure 3B showed the changes of α-helix, β-sheet, β-turn and random coil contents of MPs. In raw samples, MPs contained 50.04% α-helix, 25.15% β-sheet, 4.96% β-turn and 19.85% random coil. Compared with raw samples, the contents of secondary structures of MPs differed among the three doneness layers, indicating that the degree of doneness among different layers had a great impact on the secondary structure of MPs. This is also confirmed by Jiang et al. (32). From the inner to the outer layers, the α-helix content reduced to 38.88% in the outer layer; and the β-sheet and random coil contents improved to 30.43 and 23.96%. The α-helix reflects the orderliness of protein, which is stabilized by intrachain and interchain hydrogen bonds of peptides (19), while random coil often reflects a slacker structure. In contrast to raw samples, the α-helix content of MPs reduced greatly after heat treatment. It is speculated that the heat treatment increased the oxidation of MPs, made its secondary structure looser, and finally decreased the order and degree of its spatial structure. The inner layer contained 39.42% α-helix and 23.43% random coil, while the random coil content of middle layer increased to 24.80%. We hypothesized that the heat treatment brought about the expansion of α-helix, the generation of β-sheet and random coil, and reduced the interaction between amino acid residues. On the other side, the heat treatment of different layers may bring about the exposure of hydrophobic residues, thus increasing the hydrophobicity of MPs. The structural properties of proteins also influence the gastric and gastrointestinal phase digestibility of proteins (21). The improve of disordered structure (random coil) and the reduction of ordered structure (α-helix) are conducive to the generation of more unfolded structures. A higher degree of heat treatment decreased the α-helix content, improved the random coil content and decreased the digestibility of protein. The results showed that the outer layer underwent protein denaturation and aggregation under excessive heat treatment, thereby reducing the binding rate of protein to digestive enzymes.

The molecular simulation showed that during the heat process of 0–50 ns, the main secondary structure content of myosin in raw and cooked groups was relatively stable (Figures 3C–F). Compared with raw groups, the α-helix content of cooked groups reduced and the random coil content improved, which is consistent with Raman spectra. Therefore, the secondary structure of myosin was unfolded and expanded by heat treatment varying with the degree of doneness. The exposure of hydrophobic residues in myosin induced the unfolding of α-helix structure. To clearly explain the effect of doneness layers on the change of protein structure, we combined experiments with molecular simulation. According to the results of experiments and molecular simulation, we speculated that the middle and outer layers led to the transformation of protein structure from order to disorder, which led to the exposure of hydrophobic residues and excessive aggregation of protein denaturation, which affected the binding sites of proteins and enzymes, resulting in a decrease in digestibility.

### 3.3 Tyrosine and tryptophan changes of proteins

**FIGURE 4:**
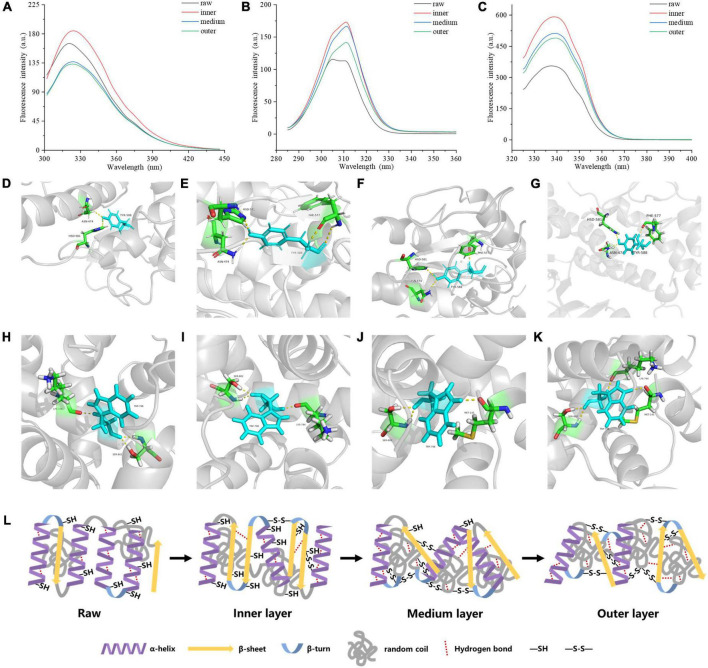
Tertiary structure of MP samples from different doneness layers: **(A)** Endogenous fluorescence spectra; **(B)** synchronous fluorescence spectra for Tyr residues (Δλ = 20 nm); **(C)** synchronous fluorescence spectra for Trp residues (Δλ = 60 nm). Hydrogen bonds between myosin aromatic amino and the surrounding amino acids treated with raw **(D,H)**, inner layer **(E,I)**, medium layer **(F,J)**, and outer layer **(G,K)**, respectively. **(L)** Mechanistic diagram of MP secondary structure, hydrogen bond and disulfide bond changes of different doneness layers.

Endogenous fluorescence is mainly derived from tryptophan (Trp) and tyrosine (Tyr), which can sensitively reflect the effect of environment on proteins, so it is generally taken into account in the study of protein structure. The content of aromatic residues exposed in a protein is reflected by the relative position of maximum fluorescence emission wavelength (λ_max_). The endogenous fluorescence λ_max_ of proteins from the three layers in cooked samples exhibited an increase from 320 to 326 nm compared with the raw samples (Figure 4A), indicating that a red shift happened in cooked samples. Energy transfer from Tyr residue to Trp residue occurred in the protein led to the fluorescence quenching of Tyr residue and the fluorescence enhancement of Trp residue. The fluorescence intensity and wavelength peak of different treatments decreased from the inner to the outer layers (Figure 4A). Figures 4B, C showed that Tyr and Trp had comparable transformations in fluorescence intensity. High fluorescence intensity reflects folded hydrophobic amino acid residues including Trp residue of meat protein. On the other hand, the lower fluorescence intensity often illustrates that the tertiary structure of a protein tends to expand partially or entirely, and the Trp residue is located in the hydrophilic environment (33).

The synchronous fluorescence results were shown in Figures 4B, C. The fluorescence of MPs in the range from 280 to 400 nm is derived from aromatic amino acids. At Δλ of 20 nm, the fluorescence spectrum just reflected the fluorescence features of Tyr residues, while Δλ at 60 nm, just the fluorescence features of Trp residues were found (34). When Δλ was set to 20 nm, the synchronous fluorescence intensities of the cooked groups were greatly stronger than those of raw groups, but the values decreased from the inner to the outer layers (Figure 4B). A red shift from 305 to 311 nm occurred on Tyr synchronous fluorescence spectra of the cooked groups compared with raw groups. When Δλ was set to 60 nm, the synchronous fluorescence spectra of MPs were shown in Figure 4C. The fluorescence intensities of the cooked groups were greatly stronger than those of raw groups, and decreased gradually from the inner to the outer layers of cooked groups, and the Trp synchronous fluorescence spectra of the cooked groups were red-shifted from 337 to 340 nm compared with raw groups. Since the λ_max_ of Tyr and Trp residues in a protein is relevant to the polarity of the environment, the conformational change of a protein can be judged according to the change of maximum emission wavelength. The synchronous fluorescence spectra of Tyr and Trp residues in MPs were separately measured at Δλ of 20 and 60 nm. From the inner to the outer layers of cooked groups, the microenvironment near the Tyr and Trp residues of MPs was changed greatly. When the polarity of MPs hydrophobic environment increases, the extension of peptide chain may increase, and more hydrophobic residues will be exposed, but the resonance energy transfer between residues and the fluorescence intensity of residues decreases (35).

As shown in Figures 4D–K, Tyr and Trp formed stable hydrogen bonds with surrounding amino acids, maintaining the stability of the protein structure. The digestibility of the outer doneness layers was lower than that of other treatments, which may be due to the improve of hydrogen bonds between Tyr and Trp residues and their surrounding amino acids (Figures 4G, K). The formation of hydrogen bonds helps to keep the stability of the protein structure (36). The number of hydrogen bonds in myosin in the outer layer was higher than that in raw samples, inner layer and middle layer, bringing about more stable structure and lower digestibility of protein.

Taken together, as the degree of doneness improved, the α-helix content in protein reduced, accompanying with increased random coil content, hydrogen bonds and disulfide bonds (Figure 4L).

### 3.4 MDS of myosin

**FIGURE 5:**
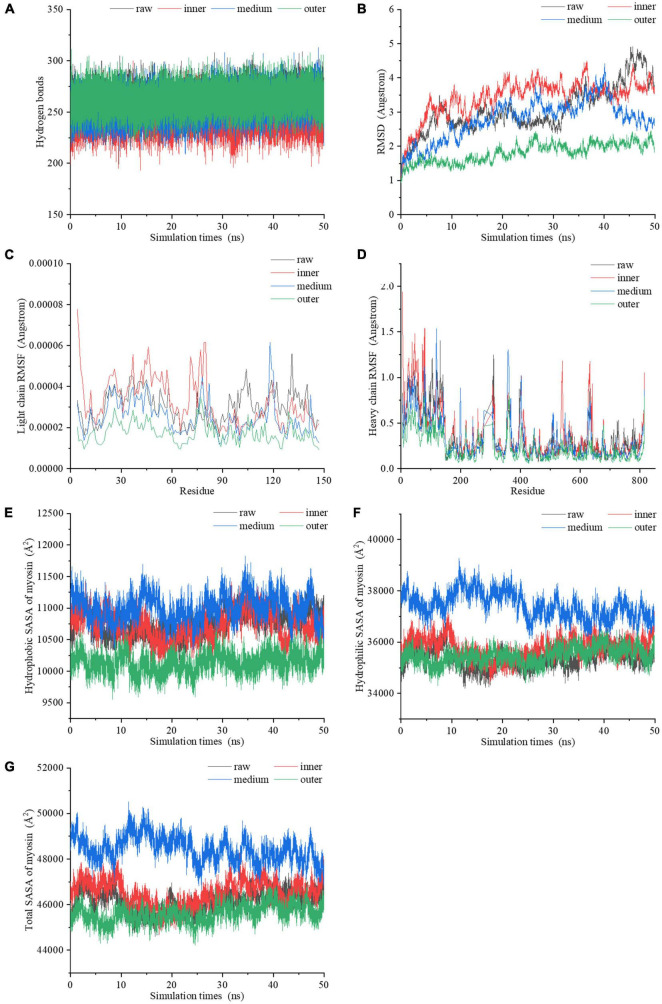
The dynamic changes of **(A)** the number of hydrogen bonds, **(B)** RMSD values, **(C)** light chain RMSF values, **(D)** heavy chain RMSF values, **(E)** hydrophobic SASA, **(F)** hydrophilic SASA, **(G)** total SASA of myosin molecules during the 50 ns MDS.

The advanced structures of proteins, such as secondary structure, tertiary structure and quaternary structure, play a vital role in the functional properties of proteins, and the existence of hydrogen bonds plays a key role in maintaining these structures (37). Hydrogen bond is the most important force to decide the secondary structure of proteins, such as α-helix, and β-sheet. The special conformation is maintained by hydrogen bonds between amino acid residues (38). Hydrogen bond is also an important interaction force between protein molecules, and proteins cannot form correct ligands without hydrogen bond. The content of hydrogen bonds in myosin was estimated to increase from the inner to the outer layers of cooked samples (Figure 5A). The results showed that as the degree of doneness improved from the inner to the outer layers, myosin was denatured and aggregated to a greater extent in the outer layer, and more hydrogen bonds were formed. This may decrease the digestibility of meat proteins in the outer layer because of the increase in the number of stable hydrogen bonds in myosin, making it more difficult to digest, and such conformations hinder the contact with the active center of digestive enzyme. As the crucial chemical force to keep the secondary structure of proteins, we hypothesized that the newly-formed hydrogen bonds to a large extent stabilized the conformation.

By comparing the root mean square deviation (RMSD) values of myosin in the simulation process, we further explored the stability of myosin structure in the process of 50 ns MDS. Figure 5B showed the RMSD changes of myosin in different heating temperature model systems. The fluctuation of molecular RMSD in myosin without heat treatment is more significant than that in heat treatment groups. In the system simulating the inner layer of cooked samples, the RMSD improved greatly in the first 5 ns, which may be relevant to the optimization of the system structure. After 5 ns, the RMSD was stable with a small increase ranging from 2.6 to 4.5 Å. In the system simulating middle layer, the RMSD increased firstly with a subsequent decline during the whole 50 ns simulation process. It is speculated that the hydrophobic residues were exposed in this process, which destroyed the structure. In the system simulating the outer layer, the RMSD values remained stable during the whole simulation process, and showed an increase from 1.0 to 2.5 Å. We hypothesized that the myosin system reached a stable state earlier because the high temperature treatment.

The root mean square fluctuation (RMSF) of molecules in MDS system can reflect the change of molecular flexibility. RMSF is calculated according to the movement deviation of different amino acid residue sites C-α in the simulation process under specific conditions. RMSF is calculated as the ratio of the final atomic position to the average position of the protein system, and is a vital indicator for the changes of the degree of protein secondary structure (39). The RMSF values of myosin light chain and heavy chain under the conditions simulating three different doneness layers fluctuated significantly during the simulation (Figures 5C, D), implying that residues had dynamic dislocation in their original positions. The experimental results showed that with the extension of holding time, the myosin molecules showed higher flexibility under the condition simulating the inner doneness layer than other conditions. The significant position of RMSF fluctuation values were basically located in the hydrophobic region, which may be due to the great change of hydrophobic group in the molecule caused by heat treatment and led to the change of protein structure. As mentioned above, we speculated that heat treatment can induce MPs aggregation, and the exposure of myosin hydrophobic residues may provide a docking site for driving the aggregation of MPs molecules.

Solvent accessible surface area (SASA), which counts on the primary and secondary structure of a protein, is a parameter for estimating the exposure of amino acids to solvents. Typically, the hydrophobic groups were hidden in the compact molecule and unfolded as the SASA value increased. In the process of simulating the heating treatment of myosin, both the hydrophobic and hydrophilic SASA curves fluctuated significantly (Figures 5E, F), indicating that the conformations of proteins changed during the simulation (40). The SASA curves of four systems (Figure 5G) showed that the secondary structure was significantly changed after heat treatment, which is compatible with the Raman spectroscopy results. Compared with values under the condition simulating the inner layer, the hydrophobic SASA of myosin under the conditions simulating the middle layer increased, indicating that the heat treatment process simulating the middle layer resulted in more hydrophobic sites being exposed on the surface of myosin. However, the hydrophobic SASA of myosin under the conditions simulating the outer layer was lower than those of the inner and middle layers, which may be due to the excessive deformation and aggregation of myosin caused by heat treatment at higher temperatures, thereby reducing the hydrophobic SASA of myosin. According to the above molecular simulation results, we found that there were significant differences in the changes of myosin in different doneness layers. We hypothesized that myosin in the outer doneness layers excessive aggregated, the active sites changed, the hydrogen bonds content increased, and the system reached a stable state earlier, all of which made the outer layers more difficult to digest.

### 3.5 Underlying mechanisms

**FIGURE 6:**
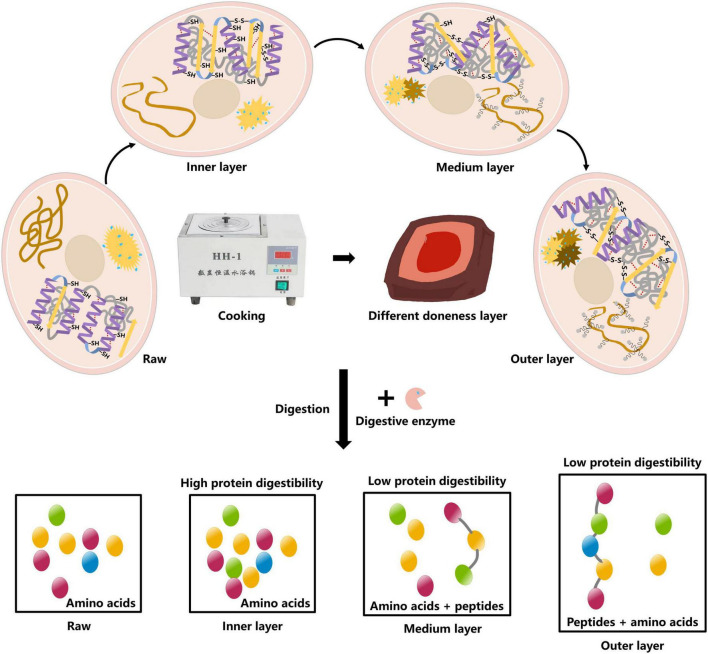
Mechanistic diagram for the effect of doneness gradient on digestibility of pork MPs.

According to the observed results, the underlying mechanism on how the degree of doneness affected protein digestibility of pork was proposed (Figure 6). As the degree of doneness increased, the α-helix in MPs gradually was transformed into random coil, and the ordered structures were destroyed, causing protein aggregation. The exposure of hydrophobic amino acid residues increased the surface hydrophobicity of proteins, and the proteins were cross-linked to form aggregations. In the middle and outer layers, MPs were thermally oxidized by forming disulfide bonds and hydrogen bonds to make the protein structure more stable and difficult to digest. We speculated that this may result in the production of indigestible peptides from the middle and outer layers.

## 4 Conclusion

During cooking, the degree of doneness improved from the inner to the outer layers of pork chops, and the functional properties of MPs (surface hydrophobicity and turbidity) increased, indicating that the partial expansion of protein molecules resulted in the exposure of hidden hydrophobic groups, but the sulfhydryl contents and solubility decreased. The destruction of secondary structure resulted in a decrease of α-helix content and an augment of random coil content. In addition, the fluorescence intensity of Trp and Tyr residues reduced from the inner to the outer layers, and the peak value was red-shifted compared with raw meat samples. The molecular simulation results showed that the heat treatment changed, to a different extent, the number of key amino acid bonds, in particular to hydrogen bonds, causing different protein digestibility from the inner to the outer layers of pork chops. The findings gave a new insight into the heterogeneity of nutritional quality of meat due to the existence of doneness gradient.

## Data availability statement

The original contributions presented in this study are included in the article/Supplementary material, further inquiries can be directed to the corresponding authors.

## Author contributions

YH: data curation, writing – original draft, investigation, formal analysis, and software. HL: data curation, software, and methodology. QL: data curation. DZ, KS, and WK: methodology. MZ: methodology and writing – review and editing. CL: conceptualization, supervision, investigation, funding acquisition, and writing – review and editing. All authors contributed to the article and approved the submitted version.
