# Supplementary material for: The degree of doneness affected molecular changes and protein digestibility of pork
Source: Front Nutr. 2023 Jan 4;9:1084779. doi: 10.3389/fnut.2022.1084779 (PMC9845567; doi:10.3389/fnut.2022.1084779)
Supplement: Supplementary file 1 [file Table_1.docx]

**The degree of doneness affected molecular changes and protein digestibility of pork**

**Yu Han^1,2,3^, Hui Liu^1,2,3^, Qian Li^1,2,3^, Di Zhao^1,2,3^, Kai Shan^1,2,3^, Weixin Ke****^1,2,3^, Miao Zhang^1,2,3*^ and Chunbao Li****^1,2,3*^**

^1^Key Laboratory of Meat Processing and Quality Control, MOE, College of Food Science and Technology, Nanjing Agricultural University, Nanjing, China,^2^Key Laboratory of Meat Processing, MARA, College of Food Science and Technology, Nanjing Agricultural University, Nanjing, China,^3^Jiangsu Collaborative Innovation Center of Meat Production, Processing and Quality Control, College of Food Science and Technology, Nanjing Agricultural University, Nanjing, China

*** Correspondences:**

Dr. Chunbao Li

E-mail: chunbao.li@njau.edu.cn

Address: College of Food Science and Technology, Nanjing Agricultural University, Weigang 1#, Nanjing, 210095, China

Dr. Miao Zhang

E-mail: t2021067@njau.edu.cn

Address: College of Food Science and Technology, Nanjing Agricultural University, Weigang 1#, Nanjing, 210095, China

***Supplementary Materials***

**2 Materials and methods**

**2.4 In Vitro Digestion**

Gastric phase digestion: The digestibility of samples was measured according to Brodkorb et al. (11) with minor modifications. Briefly, the meat samples (0.8 g) were dissolved in 3 mL simulated gastric fluid (SGF, pH 3.0), broken in the biological homogenizer (Precellys Evolution, Bertin, France) at 4 ºC, 5500 rpm for 2 × 20 s with an interval of 30 s and pre-heated at 37 ºC for 10 min. The mixture was added with 4.2 mL SGF, and the pH of the mixture was adjusted to 3.0 using 6 mol/L HCl solution, then ultra-pure water and pepsin were added (final enzyme activity:2000 U/mL) to make thegastric phase system to 10 mL. Finally, the samples were incubated at 37 ºC,200 rpm for 2 h.Twelve replicates of each sample were mixed with 10 mL of simulated intestinal fluid (SIF, pH 7.0) to endreaction by adjusting the pH to 7.0. Six replicates of each sample were used to determinegastric digestibility.

Gastrointestinal phase digestion: Then, the remaining samples were mixed with SIF, bile salts and pancreatinto arriveat a final system ratio of 1:1. In the ultimategastrointestinal system, the pH of solution was adjusted to 7.0, in whichthe trypsin activity was 100 U/mL, and bile salts concentration was 10 mmol/L. Then, the samples were incubated at 37 ºC, 200 rpm for 2 h. Two hours later, the samples were heated at 100 ºC for 5 min to stop the digestion.

**2.8 Surface Hydrophobicity**

The fluorescence probe 8-Anilino-1-naphthalenesulfonic acid (ANS) was taken to measure the surface hydrophobicity of MPs (10). An ANS solution (15 mmol/L, pH 7.0) was dissolved in PBS. ANS solution (10 μL) was mixed well with 2 mL MPs (1 mg/mL) as the treatment group. In addition, the mixture without protein was takenas the blank. Then, the mixture was kept at room temperature in the dark for 20 min.The fluorescence intensity of the mixture was taken at 370 nm as the excitation wavelength, and the fluorescence intensity was recorded from the range of 415 to 570 nm emission wavelength.

**2.12 Fluorescence Spectroscopy**

Fluorescence spectroscopy of MPs were determined according to Khan et al. (15) with minormodifications. To determine endogenous fluorescence spectra, 3 mL MPs (0.1 mg/mL) were placed in a 1 cm path quartz cell. The fluorescence spectra were taken at excitation wavelength of 280 nm, and emission wavelength of 300~450 nm for measuring the fluorescence intensity.The excitation and emission slit widths were set at 3.0 nm and 5.0 nm, respectively.To determine synchronous fluorescence spectra, the wavelength difference (Δλ) of excitation and emission of tyrosine and tryptophan residues were set at 20 nm and 60 nm, respectively. The synchronous fluorescence spectra were taken at the excitation wavelength of 265 nm. The emission wavelength of tyrosine was at a rangefrom 285 to 500 nm, while tryptophan was measured from 325 to 500 nm. Furthermore, theslit widthsof excitation and emission for Δλ_20_ and Δλ_60_ were both set to 3.0 nm.
